# Nanocarbon-based catalysts for selective nitroaromatic hydrogenation: A mini review

**DOI:** 10.3389/fchem.2022.1000680

**Published:** 2022-09-09

**Authors:** Jiarong Yao, Li Wang, Dong Xie, Linxuan Jiang, Jiantong Li, Xiaomin Fang

**Affiliations:** Henan Engineering Laboratory of Flame-Retardant and Functional Materials, College of Chemistry and Chemical Engineering, Henan University, Kaifeng, China

**Keywords:** nanocarbon, nanostructure, catalyst, nitroaromatic hydrogenation, catalytic performance

## Abstract

Selective hydrogenation of nitroaromatics to the corresponding anilines is a key topic for research in fine chemical industrial fields. Nanocarbon materials with good chemical stability, high electrical conductivity, and good mechanical performance have been regarded as promising candidates in the catalytic field, and have shown a wide range of applications in recent years. Controllable synthesis on the structure, morphology, and active sites of nanocarbon-based catalysts is vital to the development of highly efficient catalysts. In this mini-review, we summarize the recent progresses of nanocarbon materials by focusing on the synthesis approaches and their corresponding nanostructures, including carbon nanofibers, carbon nanotubes, graphene, porous carbon, carbon spheres, and metal organic framework-derived carbon materials. The design and catalytic performance of these nanocarbon materials have been systematically discussed. Finally, the emerging challenges and future prospective for developing advanced nanocarbon-based catalysts are outlined.

## Introduction

Chemical selective reduction of nitroaromatics is a critical chemical reaction process for producing amino aromatics (Cui et al., 2016; Zhang et al., 2017), which are one of the most important intermediates in the development of fine-chemicals including medicines, pesticides, dyes, and special polymer monomer (Shi et al., 2017; Duan et al., 2019; Sun et al., 2019; Wei et al., 2019; Ma et al., 2020; Feng et al., 2021).

However, the challenge for nitroaromatic hydrogenation arises from the existence of other unsaturated functional groups in one molecule, such as carbonyl, aldehyde, ester, olefin, and nitrile (Liu et al., 2016; Shi et al., 2017; Li et al., 2021; Shen et al., 2021), which are more favorably hydrogenated in thermodynamics and easily transformed into unwanted by-products (Zhang et al., 2017; Sharma et al., 2019; Shen et al., 2021). Consequently, the competitive hydrogenation leads to a decrease in selectivity for the reduction of nitroaromatics (Zhang et al., 2017; Cheong et al., 2019; Lu et al., 2022). Highly selective synthesis of target amines has become a key point for promoting the development of nitroaromatic hydrogenation technology (Lu et al., 2021; Gogoi et al., 2022).

Generally, the process of catalytic hydrogenation of nitroaromatics is as follows: nitro is first reduced to a nitroso intermediate product, and then the nitroso can be adsorbed on the surface of the catalyst for the subsequent hydrogenation reaction (Song et al., 2018; Strachan et al., 2020). Traditionally, metal-based heterogeneous catalysts are used for the synthesis of anilines from nitroaromatics (Adeyeye Nafiu et al., 2021; Shen et al., 2021). In particular, noble metal-based nanomaterials have been considered as efficient catalysts because of their excellent activity and stability (Gao et al., 2017; Yuan et al., 2018; Lu et al., 2021); however, they were limited by the low selectivity for required amines and high cost (Sun X. et al., 2018). In recent years, the non-noble metals have drawn great attention in the academic and industrial fields because of their abundance and easy availability (Zhang et al., 2022). However, the main bottleneck of their development is the harsh reaction conditions, low catalytic activity, and poor stability (Schwob and Kempe, 2016; Wei et al., 2019; Yun et al., 2019; Liu et al., 2020; Oliveira et al., 2020). To save the noble metal sources and optimize the activity of non-metallic catalysts, nanocarbon-based catalytic materials are regarded as promising candidates for next-generation catalysts and have attracted increasing attention.

**FIGURE 1:**
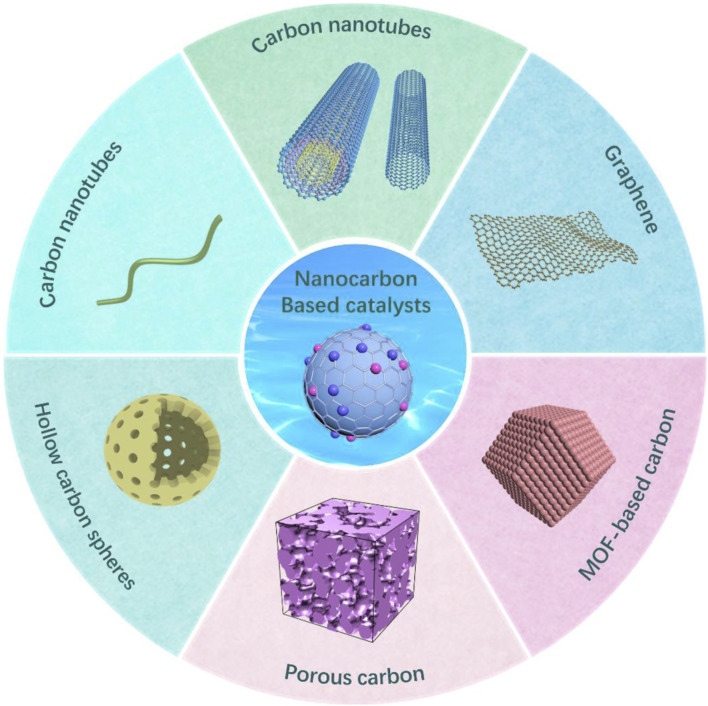
Schematic illustration of various nanocarbon-based materials for catalysts.

Nanocarbon-based materials (Figure 1) have drawn extensive attention due to their large specific surface area, high stability, and easy access to reactants (Cui et al., 2016; Sun Y. et al., 2018). It is proved that they are promising in energy conversion and storage, nano electronics, drug delivery, and biomedicine (Van Nguyen et al., 2019). In addition, they are also used as catalyst carriers; metal nanoparticles with catalytic activity loaded onto carbon-containing carrier materials can effectively reduce the amount of metal and the cost of catalysts (Chen et al., 2016). Furthermore, due to the metal having better dispersion when loaded, it has a better interaction with substrate molecules, and its catalytic activity and selectivity will be greatly enhanced (Cui et al., 2016; Gao et al., 2017; Göksu et al., 2020).

It is thus clear that the morphology and active sites of nanocarbon-based catalysts will have crucial impacts on the catalytic hydrogenation, and how to design and prepare a catalyst with various morphologies largely determines its activity and selectivity (Liu et al., 2020). At present, the common pyrolysis strategies to prepare nanocarbon-based catalysts mainly include template-assisted synthesis and template-free synthesis.

For example, catalysts with N-doped mesoporous carbon, obtained by Oliveira et al. using silica nanoparticles as templates, can be used for the catalytic hydrogenation of nitroaromatics under moderate conditions (Oliveira et al., 2020). An isolated iron single-atomic catalyst, with ordered N-doped mesoporous carbon, can be synthesized using SBA-15 as template, exhibiting excellent tolerance and activity in the hydrogenation of nitroarenes (Cheong et al., 2019). Recently, the method of template-free pyrolysis has attracted great attention because of their potential in improving the sustainability of chemical processes. Using cheap precursor materials as raw materials, carbon nanotube-encapsulated Co was prepared by using the one-pot pyrolysis method for catalytic hydrogenation and formylation of nitroarenes, and showed an exceptional catalytic activity in transfer hydrogenation (Zhu et al., 2020). In addition, a new type of polyporate material arises from the pyrolysis of metal organic framework (MOF) and has been proved to be an ideal support material for the hydrogenation of nitroaromatics (Van Nguyen et al., 2019; Yun et al., 2019). Therefore, the nanocarbon-based materials with adjustable surface properties and morphologies are more suitable supports for developing new high-performance hydrogenation catalysts.

In this mini-review, we summarize the recent advances of nanocarbon-based materials by focusing on the synthesis approaches and corresponding nanostructures, including carbon nanofibers, carbon nanotubes, graphene, porous carbon, carbon spheres, and MOF-derived carbon materials. The emerging challenges and future prospective for developing advanced nanocarbon-based materials are also outlined. We believe that this mini-review could offer some new insights and inspire extensive interests to accelerate and explore the innovations of nanocarbon-based materials.

## Carbon fiber

**FIGURE 2:**
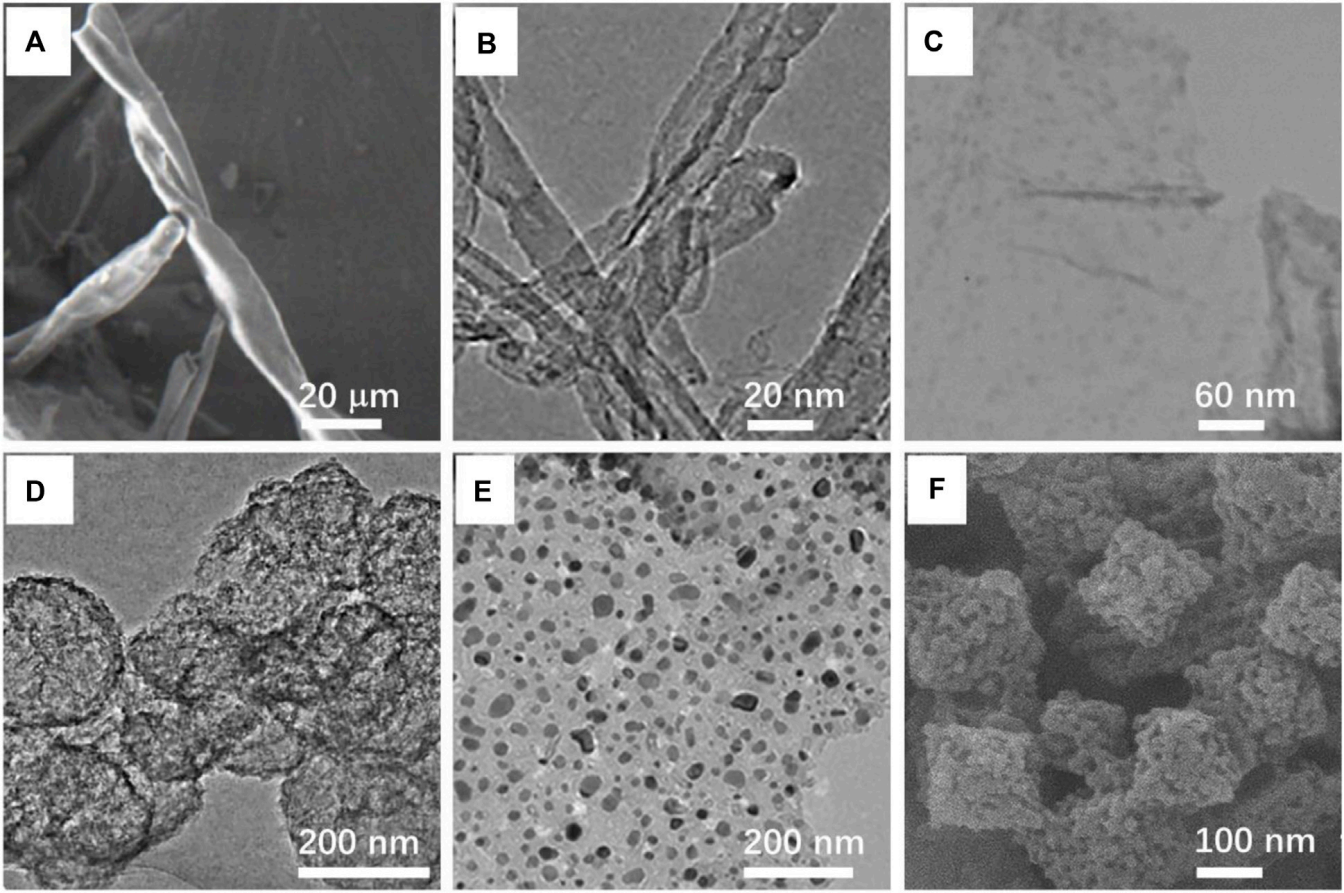
**(A)** SEM image of highly dispersed PdCo nanoparticles by modified cotton-derived carbon fibers (PdCo/CCF). Yang J. et al. (2018) with permission from the Academic Press Inc. **(B)** TEM image of phosphorus-doped carbon nanotubes. Chen et al. (2020) with permission from the American Chemical Society. **(C)** TEM image of ruthenium nanoparticles on graphene. Dabiri et al. (2021) with permission from Springer US. **(D)** TEM image of the carbon sphere templated from titaniumsilicon-1 (TS-1) zeolitethe. Huang et al. (2022) with permission from Elsevier. **(E)** TEM image of Co/Ni bimetallic nanoparticle supported by nitrogen-doped porous carbon (NPC). Shen et al. (2021) with permission from the American Chemical Society. **(F)** SEM image of the catalyst with Fe/Fe_3_C inlaid into N-doped porous carbon supports. Feng et al. (2021) with permission from Elsevier.

Owing to its high modulus, thermal stability, and high conductivity, the carbon fiber is considered as an ideal material for catalysts. Yang et al. designed a carbon fiber that used a cotton-supported metal catalyst for nitro reduction. Cotton fibers are converted into carbon fibers by carbonization in an inert atmosphere. Through a series of experimental operations, Pd and Co metal atoms are highly dispersed on the surface of the carbon fiber. Due to the ferromagnetism of cobalt, the prepared catalyst can be recycled and reused by the action of an external magnetic field, which can be used for at least ten continuous cycles without significantly reducing the catalytic activity. In addition, the catalyst realizes green catalysis by using environmentally sustainable raw materials (Figure 2A) (Yang J. et al., 2018).

Takasaki et al. reported a catalytic system of platinum nanoparticles dispersed on carbon nanofiber supports. This product can make the reaction proceed under mild conditions and keep other functional groups intact. It is an efficient and reusable catalyst for the selective reduction of nitroaromatics. At the same time, they proposed that its high chemical selectivity is mainly due to the spatial effect or electronic effect produced by the interaction between Pt particles and the carbon nanofiber carrier, rather than the size of the Pt particles (Takasaki et al., 2008).

## Carbon nanotubes

In a variety of carbon materials, carbon nanotubes with a hollow structure (Sun Y. et al., 2018), which possess high specific surface area and large surface-to-volume ratios, are widely used as catalyst supports (Göksu et al., 2020). Among these kinds of materials, the heteroatom-doped carbon nanotubes having high surface areas, appropriate price, and convenient preparation have shown a very promising application prospect (Chen et al., 2020). For instance, the P-doped carbon nanotubes (P-CNT) show good catalytic performance in various fields. Chen et al. proposed that phosphorus-doped carbon nanotubes can catalyze the reduction of nitroaromatics under mild conditions as a metal-free catalyst. The potential application of recycle P-CNT for metal-free hydrogenation is illustrated by more and more research studies (Figure 2B) (Chen et al., 2020). Similarly, the N-doped carbon nanotubes, as a reduction of the nitrobenzene catalyst, have attracted much attention. They are made to easily introduce the N atom into the carbon matrix because the sizes of the N and C atoms are closed (Li et al., 2020). Li et al. prepared a string of N-doped carbon nanotubes through the carbonization of various raw materials *in situ* polymerized pyrrole. Then, they chose a catalyst with better performance, named as NCNTs-800, to test the performance (Li et al., 2020). Chi et al. synthesized N-doped carbon nanotubes by the pyrolysis of chitosan-CNT composite. The chitosan was used as the nitrogen source because of its cheap price, rich storage, and harmlessness (Chi et al., 2021).

Multi-walled carbon nanotubes are a kind of carbon nanotube materials that have a unique hollow structure and good conductivity (Chi et al., 2021). Göksu et al. reported multi-walled carbon nanotube-supported palladium/copper nanoparticles. This catalyst which has bimetallic nanoparticles and excellent supported carbon material showed some unexpected physicochemical properties. Actually, the performance of noble metal catalysts is also in line with their expectations, but the use is limited by high price and hard availability. The application will be promoted by using special support such as carbon-based materials which can decrease the high cost and increase the metal dispersed. The nitroaromatic compounds used by the catalyst can be changed into target products in a yield of more than 99% within 10 min at room temperature (Göksu et al., 2020).

Sun et al. fabricated three-dimensional graphene/carbon nanotube hybrid catalysts. Due to the synergistic effect of the carbon nanotubes and graphene, the catalysts they synthesized showed high stability and high activity. By mixing melamine and nickel compounds, they synthesized the target catalyst by pyrolysis. Because a large number of nickel atoms coated with carbon film were dispersed on the carbon nanotube chamber and the surface of graphene, the catalyst showed satisfactory performance (Sun Y. et al., 2018).

## Graphene

The graphene, a two-dimensional single-layer film of sp^2^ hybrid-carbon, has a large surface area and can be used as a catalyst carrier to immobilize and stabilize metal nanoparticles (Jagadeesh et al., 2015). It is reported that graphene can be used as a useful helper to provide active centers in nanocomposites and reduce the accumulation of nanoparticles (Huang et al., 2012).

Das et al. made a magnetic recyclable catalyst containing silver by loading graphene oxide with Fe_2_O_3_ and silver oxide particles, and with the formation of magnetite nanoparticles, partially reduced graphene oxide was produced. The catalyst can be used in the hydrogenation of nitroaromatics to amino aromatics with 2-propanol as the hydrogen source. Except for the necessary intermediate azobenzene, no other intermediate products were detected in this reaction (Das et al., 2018). Noble metal catalysts have been vigorously replaced, which will promote the development of non-noble metal catalysts with higher selectivity and stability. However, nanoparticles are not air-stable and will be inactivated rapidly in practice (Chen et al., 2016). In order to solve this problem, Chen et al. coated the cobalt oxide precursor with a nanographite material. Through further heating treatment, a part of the cobalt oxide was reduced to a zero-valent metal state, which showed catalytic activity, and the nano graphite layer can be used as the source of carbon to prepare a graphene layer, which showed excellent recyclability and satisfactory performance in ten-cycle experiments (Chen et al., 2016).

The disadvantage of graphene is that they are difficult to separate and recycle. To address this issue, researchers propose that three-dimensional porous graphene with high specific surface area, low density, and strong mechanical strength can be used (Li and Shi, 2012). With graphene oxide and RuCl_3_ as the reactants, Dabiri et al. successfully prepared Ru nanoparticles on nitrogen-sulfur-doped three-dimensional graphene by using the one-pot hydrothermal method. The catalyst showed excellent recoverability in the selective reduction of nitroaromatics to the corresponding aniline (Figure 2C) (Dabiri et al., 2021). Zhang et al. fabricated nitrogen-rich carbon materials by hydrothermal co-assembly of two-dimensional graphite carbon nitride and graphene. Then, palladium nanoparticles were fixed on the resulting material by impregnation. This heterogeneous catalyst uses formic acid as the hydrogen source to convert nitroaromatics to aniline, which has good conversion and high selectivity (Zhang et al., 2020).

## Carbon spheres

Hollow carbon spheres can be considered as excellent catalyst supports because of their large void fraction, low effective density, and large specific surface area (Sun et al., 2013). Sun et al. developed a method of encapsulation of magnetic Fe_3_O_4_ nanoparticles within the hollow core and embedded platinum nanoparticles in the carbon shell for the preparation of bifunctional magnetic yolk-shell-type nano-catalysts. This product has magnetic response characteristics because of the existence of Fe_3_O_4_; the separation can be realized under the action of a magnet, which solves the problem of recycling the nano-catalyst. Carbon shells were used as noble metal catalyst supports supported with Pt nanoparticles. The nanocomposites had high catalytic activity, reusability, and good magnetic separation performance in nitrobenzene hydrogenation (Sun et al., 2013). Sadjadi et al. used glucose-derived carbon as a shell, which was embodied in nitrogen-doped porous carbon, to coat magnetic nanoparticles as a material matrix, and then fixed with palladium nanoparticles on its surface to obtain a magnetic catalyst. The nitrogen-rich porous carbon shell can provide higher palladium loading and inhibit the leaching of palladium, thus showing high catalytic activity. Adding palladium before carbonization can obtain higher palladium loading than after heat treatment (Sadjadi and Heravi, 2019).

Compared with some microporous carbon materials, porous carbon spheres have the unique characteristics of regular geometry, large specific surface area, adjustable porosity, and particle size (Sun et al., 2013; Yang et al., 2018e). Based on these advantages, porous carbon spheres have been used as a promising material for adsorption, heterogeneous catalysis, and energy storage (Yang et al., 2018e). Huang et al. synthesized nanoporous carbon with a hollow sphere structure by chemical vapor deposition using titanium-silicalite as the template. By the test results, the catalytic activity and selectivity of the platinum catalyst on nanoporous carbon for nitrobenzene hydrogenation were significantly higher than those on activated carbon because of the platinum particles having high porosity and being well dispersed (Figure 2D) (Huang et al., 2022).

In addition to the porous carbon materials mentioned previously, the Co-doped graded carbon spheres show better catalytic performance because they can provide higher pore volume and rich active centers. Yang et al. synthesized graded porous carbon spheres of Co doped with nitrogen, phosphorus, and sulfur (denoted as NPs-HPCs). The results showed that porous carbon spheres are a good carrier for anchoring highly dispersed and uniform noble metal nanoparticles for heterogeneous catalysis, consequently leading to an excellent catalytic performance for the reduction of nitroaromatics (Yang et al., 2018e).

## Porous carbon

Porous carbon materials with large surface area, porosity, and rich surface chemical properties, which are widely used in many fields, are applied as supporting materials to produce catalysts with high selectivity and high conversion.

Recently, it has been reported that heteroatom doping of porous carbon materials is a significant method to further change the energy stripe structure and electron transfer characteristics, along with the surface polarity of carbon-based materials. Moreover, supported noble-metal-based catalysts that exhibit high catalytic activities have been widely used in transforming nitroarenes to aminoarenes. In addition, supported noble-metal-based catalysts are often accompanied with high cost and have inherent low selectivity. Hence, in the past decade, exploring heteroatom-doped porous carbon materials loaded with non-noble metals as new nitro reduction catalysts has become an exciting topic in the field of catalysis.

Many efforts have been paid attention to develop the nitrogen-doped carbon materials in recent years due to the N atoms creating more structural defects by enriching the protons on the surface of the modified carbon (Wang et al., 2017).

Yun et al. developed Fe-based NPs as an N-doping porous carbon stemming from pyrogenic decomposition melamine to make a production of efficient catalytic transfer hydrogenation active sites. This iron-based catalyst can be reused at least six times without losing any activity (Yun et al., 2019). Cheong et al. prepared an ordered mesoporous Fe1/N−C catalyst. It showed high activity and chemical selectivity in the transfer hydrogenation of nitroaromatics to aromatic amines (Cheong et al., 2019). Oliveira et al. presented a tactic of synthesizing N-doped mesoporous carbon and the immobilization of sub nanometer-dispersed cobalt catalyst in mesoporous nitrogen-doped carbon matrix, showing a remarkable activity and selectivity (Oliveira et al., 2020).

In addition to loading a single metal atom, the synergistic effect of different metal atoms will have a better catalytic effect on the reaction. Liu et al. fixed Co and Cu bimetals on nitrogen-mingled porous carbon through a significant ligand stable pyrolysis tactic. And this material exhibited unexpected catalytic properties (Liu et al., 2020). Shen et al. designed a new novel Co/Ni bimetal nanoparticle supported by nitrogen-doped porous carbon (Co_5_/Ni@NPC-700), which showed a catalytic conversion of nearly 100% and a chemical selectivity of more than 98% (Figure 2E) (Shen et al., 2021).

In addition, some experiments have proved that when two strange atoms are doped into porous carbon materials together, there can be a cooperative effect between the heteroatom dopants. Compared with single atom-doped porous carbon materials, the catalytic properties of the materials have been significantly improved.

Duan et al. prepared single-phase pyrite FeS_2_ nanoparticles by a simple and economical method with porous carbon co-doped N and S as carriers. Under mild water reaction conditions, the synthesized FeS_2_/NSC has preferential adsorption on nitro. Thus, the catalytic hydrogenation of various functionalized nitroaromatics with broad-spectrum functional groups has excellent catalytic activity and unique selectivity, and can be recycled for up to eight times (Duan et al., 2019).

Coincidentally, the synthesis of diatomic-doped carbon porous carbon materials from non-carbon materials also shows a good trend. Wei et al. fabricated O, N, Co-doped porous carbon (ONPC) from coal tar and residue asphaltene. The preparative ONPC has a heavy specific surface area and prosperous aperture gap structure. As a metal-free carbon catalyst, it provides rich carbon positive centers, effectively improving its catalytic energy, and has strong durability for the rundown of nitrobenzene to aniline (Wei et al., 2019).

## MOF-derived carbon

In the field of catalysis, MOF materials have attracted extensive attention because of their rich elements, large specific surface area, and adjustable pore size (Figure 2F) (Yang et al., 2018d; Feng et al., 2021). Numerous researchers used the MOF as a precursor material in the design of catalysts for hydrogenation of nitroarenes (Yan et al., 2021). Coating metal ions with carbon-based materials has become a widely used selective catalyst because the completely exposed metal particles do not have this attribute (Shaikh et al., 2021). As a catalyst for the reaction, it is very important that products should easily separate from the surface of the catalyst (Yang et al., 2018d). Compared with nanocarbon-loading noble metal ion catalysts with high cost, these nanocarbon loading non-noble metal ions are preferable to be discussed in catalytic hydrogenation.

In 2018, Yang et al. first reported a MOF-derived cobalt sulfide catalyst which can be used for the selective reduction of nitroarenes. This catalyst did not have the common shortages of Pd-based catalysis and showed outstanding activity and chemo-selectivity. In addition, the reduction of nitroarenes not only had mild reaction conditions, but was also separated by simple steps which can reduce the wasted organic solvent to achieve sustainable chemical processes (Yang et al., 2018c).

Shaikh et al. used the N-containing MOF as a resource of both the active metal catalyst and nitrogen-rich graphitic carbon loaded on a magnetic Fe_3_O_4_ crystal to synthesis the Co_3_O_4_/nitrogen-doped graphitic carbon/Fe_3_O_4_ nanocomposites. This catalyst has a lot of advantages such as being reusable, highly active, and cost-effective to selective catalytic hydrogenation of nitroarenes under mild conditions (Shaikh et al., 2021).

In 2017, Yang et al. showing a cobalt phosphide in catalyze nitroarenes hydrogenation which used red-phosphorous infused with ZIF-67 nanocubes as the precursors and then pyrolysis of the production. There are a wide range of sources of activated cobalt phosphides for various reactions. The reaction using this catalyst can be carried out at a lower temperature than other metal-based catalysts (Yang et al., 2018b).

Compared with the previous cobalt nanoparticles/molecules, Wang et al. reported cobalt single-atoms supported on an N-doped carbon catalyst which showed better catalytic performance because of their unique features such as excellent tailorability, good designability, clear porous structure, and ultra-high specific surface area. The reason why this catalyst has had such an excellent performance is that it combines the size effect and solvent effect that nitrobenzene can be reduced in a water/ethanol mixed-solvent, and the selectivity of aniline can be increased to 99.1% (Wang et al., 2020).

## Discussion

Carbon nanomaterials have good conductivity, high specific surface area, and good stability. In this article, several catalysts for selective reduction of nitroaromatics based on carbon nanomaterials are reviewed, such as porous carbon, carbon nanotubes, graphite, carbon spheres, carbon nanofibers, and MOF-derived carbon materials. In addition, we also put forward the characteristics of various carbon-based materials as catalyst supports. Based on the aforementioned analysis, we believe that the nitro selective reduction catalyst supported on carbon nanomaterials will present the following aspects in the future research: 1) Research on catalysts for nitro hydrogenation under mild conditions and for industrial mass production; 2) the recoverability of the catalyst is also a key point that must be considered in future research, for example, how to introduce magnetic species on carbon while achieving enhanced catalytic performance; and 3) the mechanism of competitive adsorption between the support and solvent on nanocarbon-based catalysts for catalytic hydrogenation should been further studied, which provides a theoretical basis for the design of hydrogenation catalysts.
